# Multi-scale Entropy Evaluates the Proarrhythmic Condition of Persistent Atrial Fibrillation Patients Predicting Early Failure of Electrical Cardioversion

**DOI:** 10.3390/e22070748

**Published:** 2020-07-07

**Authors:** Eva María Cirugeda Roldan, Sofía Calero, Víctor Manuel Hidalgo, José Enero, José Joaquín Rieta, Raúl Alcaraz

**Affiliations:** 1 Research Group in Electronic, Biomedical and Telecommunication Engineering, University of Castilla-La Mancha, 16071 Cuenca, Spain; eva.cirugeda@uclm.es; 2 Cardiac Arrhythmia Department, University Hospital of Albacete, 02006 Albacete, Spain; sofia.calero@uclm.es (S.C.); victor.h@uclm.es (V.M.H.); j.n.ero@uclm.es (J.E.); 3 BioMIT.org, Electronic Engineering Department, Universitat Politécnica de Valencia, 46022 Valencia, Spain; Jose.rieta@upv.es

**Keywords:** atrial fibrillation, electrocardiogram, electrical cardioversion, sample entropy, multiscale entropy, composite multiscale entropy, refined multiscale entropy

## Abstract

Atrial fibrillation (AF) is nowadays the most common cardiac arrhythmia, being associated with an increase in cardiovascular mortality and morbidity. When AF lasts for more than seven days, it is classified as persistent AF and external interventions are required for its termination. A well-established alternative for that purpose is electrical cardioversion (ECV). While ECV is able to initially restore sinus rhythm (SR) in more than 90% of patients, rates of AF recurrence as high as 20–30% have been found after only a few weeks of follow-up. Hence, new methods for evaluating the proarrhythmic condition of a patient before the intervention can serve as efficient predictors about the high risk of early failure of ECV, thus facilitating optimal management of AF patients. Among the wide variety of predictors that have been proposed to date, those based on estimating organization of the fibrillatory (*f*-) waves from the surface electrocardiogram (ECG) have reported very promising results. However, the existing methods are based on traditional entropy measures, which only assess a single time scale and often are unable to fully characterize the dynamics generated by highly complex systems, such as the heart during AF. The present work then explores whether a multi-scale entropy (MSE) analysis of the *f*-waves may provide early prediction of AF recurrence after ECV. In addition to the common MSE, two improved versions have also been analyzed, composite MSE (CMSE) and refined MSE (RMSE). When analyzing 70 patients under ECV, of which 31 maintained SR and 39 relapsed to AF after a four week follow-up, the three methods provided similar performance. However, RMSE reported a slightly better discriminant ability of 86%, thus improving the other multi-scale-based outcomes by 3–9% and other previously proposed predictors of ECV by 15–30%. This outcome suggests that investigation of dynamics at large time scales yields novel insights about the underlying complex processes generating *f*-waves, which could provide individual proarrhythmic condition estimation, thus improving preoperative predictions of ECV early failure.

## 1. Introduction

Atrial fibrillation (AF) is the most commonly encountered cardiac arrhythmia in clinical practice, nowadays affecting more than 33 million people worldwide [1]. Nonetheless, its prevalence is closely related to aging, and it is hence expected to triple in the next four decades [2]. During the arrhythmia, the electrical excitation process of the atria and their subsequent mechanical activity are extremely rapid and uncoordinated, thus leading to an ineffective atrial pumping function. While this condition is not life-threatening in itself, it provokes hemodynamic alterations predisposing to the formation of blood clots and then increasing the likelihood of triggering a critical stroke [3]. In fact, AF patients present a five-fold risk of stroke and a two-fold risk of death compared to healthy people of the same age [4]. Moreover, 20% of total strokes approximately occur in patients suffering from this arrhythmia [5]. Furthermore, regardless of stroke, AF has been associated with cognitive decline and vascular dementia [6], as well as both reduced exercise capacity and quality of life [7].

In many patients the arrhythmia exhibits a progressive nature, being commonly classified into four major groups according to its course, i.e., paroxysmal, persistent, long-standing persistent, and permanent AF [8]. Paroxysmal AF is the first manifestation of the disease and shows self-terminating episodes lasting for less than seven days. Persistent AF is characterized by episodes lasting for more than a week and requiring an external intervention for its termination. About 25% of patients suffering from intermittent paroxysmal AF episodes evolve to a persistent stage in less than five years [9]. When the arrhythmia lasts for more than a year, it is called long-standing persistent AF. Finally, when it is impossible to restore sinus rhythm (SR), both the patient and the clinician make a joint decision to avoid more interventions for that purpose and AF is labelled as permanent [8].

While epidemiological studies have found cardiovascular complications even in patients presenting only brief paroxysmal AF episodes, significantly higher risk of stroke and mortality have been reported for those suffering from persistent or permanent arrhythmias [10]. This finding has led to strongly recommend the use of early and effective interventions for mitigating AF progression [11]. For that purpose, current clinical guidelines for the management of AF underline electrical cardioversion (ECV) as the method of choice in hemodynamically compromised patients with new-onset AF [12]. Furthermore, in hemodynamically stable patients ECV has also shown some advantages regarding the other common treatment to restore SR in initial stages of AF, i.e., pharmacological cardioversion. Indeed, ECV is able to restore SR quicker and more effectively, then reducing hospitalization time [13]. It is therefore not surprising the recent trend towards ECV in emergency departments to return AF patients back to SR [14].

The procedure of ECV consists of delivering one or more synchronized transthoracic electrical shocks to the patient until SR is restored or the maximum allowed shock voltage is achieved [15]. Before ECV, patients are often treated with antiarrhythmic drugs, since they increase the probability of restoring SR and reduce immediate AF recurrence [12,16]. However, although SR is initially restored in more than 90% of the patients, AF recurrence is common during the follow-up, even using potent antiarrhythmic drugs. Thus, about 20–30% of patients relapse to AF within one month, 40–60% within three months, and 60–80% in less than a year [15,17,18]. Within this context, identification of patients with an increased risk of early AF recurrence is important for a rational clinical therapeutic strategy [19]. Thus, the procedure could be avoided on those patients with high chance of early failure due to their proarrhythmic condition, saving healthcare costs and preventing them from associated risks and side effects. While ECV rarely provokes major complications, some collateral effects include sedation-related hassles, hypotension, post-shock bradycardia, malignant ventricular arrhythmias, or arterial thromboembolism [15].

So far, a variety of studies have evaluated common clinical variables and risk factors in the patient to anticipate ECV outcome, such as age, arrhythmia duration, and presence of vascular or coronary diseases [18,20]. Similarly, other works have analyzed echocardiographic variables, including left atrium volume, right atrium area, ejection fraction, left appendage size, and conduction velocity in left appendage [21,22,23]. However, these indices have not been strongly predictive or are alternatively difficult to measure in clinical practice without very specialized devices [24]. To palliate these problems, some parameters estimated from the easily accessible, cheap, and non-invasive surface electrocardiogram (ECG) recording have also been proposed. These metrics are mainly based on quantifying the signs provoked by AF on the ECG, such as the replacement of regular P-waves by other waveforms of different sizes, amplitude and timings, which are named fibrillatory (*f*-) waves, as well as the development of a rapid and irregular ventricular response [8]. Indeed, indices assessing RR irregularity [25,26,27,28,29], QRS fragmentation [30], and morphology of the *f*-waves [24,31,32,33,34,35,36] can today be found in the literature.

Among these parameters, those estimating organization and regularity of *f*-waves through common entropy-based metrics have reported very promising results [36,37]. However, these traditional entropy measures only evaluate a single temporal scale, and sometimes are not able to fully characterize all the dynamics generated by highly complex physiological systems [38]. Hence, the main goal of the present study is to explore whether a multi-scale entropy (MSE) analysis applied to the *f*-waves can provide an improved proarrhythmic condition estimation, thus yielding better preoperative predictions about ECV outcome in persistent AF patients. MSE was initially proposed by Costa et al. [38] as an extension of the well-known Sample Entropy (SE) to estimate the complexity of a time series in a wide-range of temporal scales. While this approach has been broadly used to characterize different physiological signals, some modifications have also been proposed to overcome its limitations [39]. Hence, in addition to the common MSE, its composite (CMSE) [40] and refined (RMSE) [41] versions will also be analyzed in the present work.

The remainder of this paper is organized as follows. Section 2 describes the study population, as well as the procedures of acquisition and preprocessing of ECG recordings to remove noise and extract the *f*-waves. Moreover, some previously proposed predictors of ECV outcome, along with other novel parameters extracted from the MSE analysis of the *f*-waves are also introduced in this section. Classification results between patients who relapsed to AF and maintained SR after the follow-up are next presented in Section 3 and discussed in Section 4. Finally, Section 5 presents the concluding remarks of this study.

## 2. Materials and Methods

### 2.1. Study Population

Seventy patients diagnosed of persistent AF (32 men and 38 women), under treatments with antiarrhythmic and anticoagulation drugs, and indicated for ECV were enrolled in the study. All patients gave their consent and underwent the cardioversion procedure at University Hospital of Albacete (Spain). The study was approved by the Ethical Review Board of this institution. All cardioversions were performed with anesthesiology assistance under general sedation. For the application of synchronized electrical shocks, one paddle was firmly placed in the second intercostal space on the right side parasternally and the other one was located in a left-sided lateral position along the midaxillary line [37]. The electrical energy used for cardioversion followed the increasing sequence of 200, 300, 360 and 360 J, with a maximum number of four electrical shocks. The procedure was initially successful in all patients, who recovered and maintained SR until hospital discharge (two hours later). After a follow-up of four weeks, 31 patients maintained SR and the remaining 39 relapsed to AF. During this time, all patients received anticoagulants and antiarrhythmic drugs by clinical judgment. Table 1 provides more clinical information about the study population.

### 2.2. Acquisition and Preprocessing of the ECG Signal

A standard 12-lead ECG signal was continuously recorded from each patient some minutes before ECV and during the entire procedure. The recording was acquired with a sampling rate of 1024 Hz and 16-bit of resolution over a dynamic range of ±5 mV. A 90 s-length interval extracted just before the first electrical shock from lead V1 was analyzed. This lead was selected because it typically exhibits *f*-waves with the largest amplitude [42]. The segment was preprocessed for removal of baseline wander, powerline interference, and high frequency noise. More precisely, baseline wander was estimated using a 3rd order Butterworth low-pass filtering with cut-off frequency of 0.8 Hz and then subtracted from the original signal [43]. A backward-forward IIR filtering was chosen for preservation of phase and amplitude characteristics, as the Butterworth filter shows a maximally flat response in the bandpass. Next, the powerline interference was removed by means of a notch filter with central frequency of 50 Hz and bandwidth of 4 Hz [43]. Finally, a low-pass filtering with cut-off frequency of 70 Hz was used to remove high-frequency noise [43].

**Figure 1:**
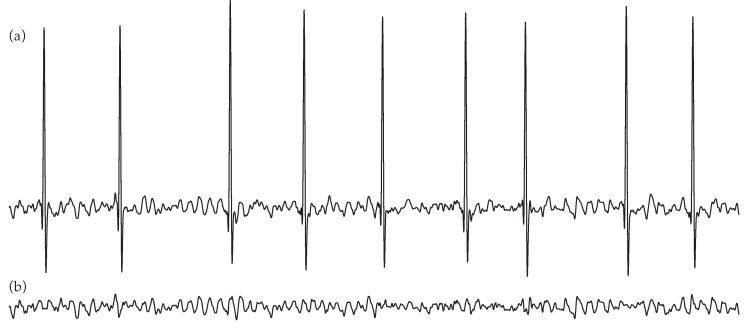
Example of a common electrocardiogram (ECG) segment (**a**), along with the extracted *f*-waves (**b**) by average QRST complex subtraction.

To reliably analyze the *f*-waves, they were firstly extracted from the preprocessed ECG by making use of a well-established QRST cancellation method [44]. In brief, R-peaks were detected using a previously published algorithm [45], and ectopic beats were identified according to a template matching approach [46,47]. Next, the QRST complex length was experimentally set to the minimum between its common duration (i.e., 470 ms) and 90% of the median RR interval. Then, all complexes were delineated by positioning that window centered on R-peaks. If ectopic beats were found, they were averaged to obtain a template for their cancellation [48]. Similarly, another template was computed by averaging the beats labelled as normal. After cancelling all normal and ectopic complexes, the resulting signal was high-pass filtered with a cut-off frequency of 3 Hz to obtain *f*-waves as clean as possible from baseline wander and QSRT residua [36]. As an example, Figure 1 shows the *f*-waves obtained from a typical ECG interval.

### 2.3. Methods for Predicting Electrical Cardioversion Outcome

As a reference, some metrics well-established in the literature to anticipate AF recurrence after ECV were firstly computed from the *f*-waves. Thus, amplitude and normalized amplitude of these waveforms (FWA and nFWA), along with their dominant frequency (DF), were obtained [49]. Moreover, regularity of the *f*-waves was estimated via SE. Next, a multi-scale entropy analysis was applied to the *f*-waves by considering three algorithms, such as MSE, CMSE, and RMSE. While a 90 s-length ECG interval was analyzed for each patient, it was divided into three non-overlapped segments, and the metrics were computed for each one and averaged. More details are provided in the following subsections.

#### 2.3.1. Existing Predictors Analyzing the *f*-Waves

FWA and nFWA were automatically computed as in previous works [37]. More precisely, naming the preprocessed ECG recording as x(n) and *f*-waves as f(n), both signals having a length of *N* samples, these parameters were estimated as [37]

(1) $$FWA\left(f,N\right)=\sqrt{\frac{1}{N}\sum_{n=1}^{N}{\left|f\left(n\right)\right|}^{2}},\;\mathrm{and},$$

(2) $$nFWA\left(f,x,N,N_{b}\right)=\frac{\sqrt{\frac{1}{N}\sum_{n=1}^{N}{\left|f\left(n\right)\right|}^{2}}}{\sqrt{\frac{1}{N_{b}}\sum_{k=1}^{N_{b}}{\left|x\left(k\right)\right|}^{2}}},$$

where *k* indexes R-peaks, and $N_{b}$ is the number of beats in the signal.

To obtain the DF, power spectral density (PSD) of the *f*-waves was computed using the Welch periodogram. A Hamming window of 6.144 points in length, a 66% overlapping between adjacent windowed sections, and a 6.144-point fast Fourier transform were used as computational parameters. Nonetheless, it should be noted that those windows whose PSD exhibited a cross-correlation with the remaining ones lower than 0.7 were discarded. In this way, confounding effect of intervals corrupted by excessive noise or QRST residua on DF computation was avoided. Finally, the DF was estimated from the averaged PSD as the frequency corresponding to the largest amplitude within the 3–12 Hz band [50].

On the other hand, organization of the *f*-waves was assessed by computing SE from their main component, such as in [37]. This fundamental waveform, referred to as ff(n), was obtained by filtering *f*-waves with a 9th order IIR Chebyshev type 2 structure, centered on the DF with a bandwidth of 5 Hz and an attenuation of 20 dB in the stop band [51]. It is well-known that SE evaluates self-similarity within a non-stationary time series, larger values suggesting more irregularity and disorganization [52]. From a mathematical point of view, computation of this entropy relies on finding similar patterns between different epochs of length *m* separated by a distance *r* along the signal, and its probability of maintenance when the length of the epochs *m* is increased by one unit [52]. More precisely, given the signal ff(n), the algorithm is as follows [52]:

1. Form N−m vectors $v_{m}\left(j\right)$ of length *m* samples, such that
  (3) $$v_{m}\left(j\right)=\left\{ff(j+i):\;1\leq j\leq N-m,\;\;0\leq i\leq m-1\right\}.$$
2. Compute the Chebyshev distance between every pair of vectors, i.e.,
  (4) $$\begin{array}{cc} d_{jk}\left(m\right) & =d\left\{v_{m}\left(j\right),v_{m}\left(k\right)\right\}\\ \; & =\max\left\{|v_{m}\left(j\right)-v_{m}\left(k\right)|\right\}\\ \; & =\max_{i}\left\{|ff(j+i)-ff(k+i)|:\;0\leq i\leq m-1\right\}. \end{array}$$
3. Estimate the number of matches of length *m* for every vector $v_{m}\left(j\right)$. A match is obtained when the dissimilarity distance, $d_{jk}\left(m\right)$, is below a threshold *r*.
  (5) $$B_{j}^{m}\left(r\right)=\frac{1}{N-m-1}\sum_{\begin{array}{c} k=1\\ k\neq j \end{array}}^{N-m}\left(d_{jk}\left(m\right)<r\right).$$
4. Define the probability that two sequences of *m* points will match as
  (6) $$B^{m}\left(r\right)=\frac{1}{N-m}\sum_{j=1}^{N-m}B_{j}^{m}\left(r\right).$$
5. Increase the sequence length to m+1 and repeat steps 1 to 4 to obtain
  (7) $$A_{j}^{m}\left(r\right)=\frac{1}{N-m-1}\sum_{\begin{array}{c} k=1\\ k\neq j \end{array}}^{N-m}\left(d_{jk}\left(m+1\right)<r\right),\;\mathrm{and}$$
  (8) $$A^{m}\left(r\right)=\frac{1}{N-m}\sum_{j=1}^{N-m}A_{j}^{m}\left(r\right).$$
6. Finally, estimate SE as [52]
  (9) $$SE\left(ff,N,m,r\right)=-\ln\frac{A^{m}\left(r\right)}{B^{m}\left(r\right)}.$$

While SE is completely dependent on the parameters *m* and *r*, no clear rules exist for selecting their optimal values. After some experiments, the widely recommended values of m=2 and r=0.2 times the standard deviation of ff(n) were chosen in the present study [53].

#### 2.3.2. Novel Predictors from Multi-Scale Entropy Analysis of the *f*-waves

Generally, time series derived from complex systems are likely to present structures on multiple spatiotemporal scales, and hence SE may be unable to completely capture their dynamics. To palliate this issue, Costa et al. proposed MSE as an extension of SE where this entropy is computed over several coarse-grained versions of the original time series [38,54]. Thus, in the present work the fundamental component of the *f*-waves was firstly decomposed into different coarse-grained time series [38], i.e.,

(10) $$ff^{\tau }\left(n\right)=\frac{1}{\tau }\sum_{l=0}^{\tau }ff\left(n\tau -l\right),\;\mathrm{for}\;1\leq n\leq \frac{N}{\tau },$$

and τ being the scale factor. Next, SE was computed for each scale according to the previously described approach and remaining m=2 and r=0.2 times the standard deviation of ff(n). Thus, MSE was obtained as [38]

(11) $$MSE\left(ff,N,m,r,\tau \right)=SE\left(ff^{\tau },\frac{N}{\tau },m,r\right).$$

It should be noted that $ff^{\tau }\left(n\right)$ was obtained by averaging non-overlapped windows of τ samples, thus decreasing the length of each coarse-grained time series by a factor τ. In this way, the variance in entropy estimates increases when τ also grows, and imprecise measurements could be obtained for large time scales. To overcome this limitation, CMSE has been proposed by Wu et al. [40]. In this approach, entropy is computed for each scale by averaging τ values obtained from τ coarse-grained times series, which are obtained as [40]

(12) $$ff_{k}^{\tau }\left(n\right)=\frac{1}{\tau }\sum_{l=0}^{\tau }ff\left(n\tau -l+k\right),\;\mathrm{for}\;1\leq n\leq \frac{N}{\tau },$$

and *k* ranging from 0 to τ−1. Then, CMSE is estimated as [40]

(13) $$CMSE\left(ff,N,m,r,\tau \right)=\frac{1}{\tau }\sum_{k=0}^{\tau -1}SE\left(ff_{k}^{\tau },\frac{N}{\tau },m,r\right).$$

However, MSE and CMSE still present two major shortcomings. On the one hand, although coarse- grained time series are obtained by decimation, aliasing errors are not avoided and spurious oscillations could occur in these rescaled signals. On the other hand, the threshold *r* remains constant for all time scales, thus provoking an artificial reduction in values of entropy for large scales. To mitigate these constraints, Valencia et al. [41] proposed a refined version of MSE, i.e., RMSE. This new method is mainly based on eliminating the fast temporal oscillations before downsampling the original time series. For this purpose, a 6th order low-pass Butterworth filter with a normalized cut-off frequency of 0.5/τ was used in the present work [41]. Backward-forward IIR approach was considered to perform a zero-phase filtering [41,43]. Moreover, the threshold *r* was taken as 0.2 times the standard deviation of the filtered times series for each scale. More precisely, RMSE was computed as [41]

(14) $$RMSE\left(ff,N,m,r,\tau \right)=SE\left({\hat{ff}}^{\tau },\frac{N}{\tau },m,\hat{r}\right),$$

where ${\hat{ff}}^{\tau }$ was the filtered and coarse-grained time series for each scale and $\hat{r}$ was the dissimilarity distance specifically obtained from ${\hat{ff}}^{\tau }$.

To obtain reliable SE estimates, previous works have recommended that *N* should be at least $10^{m}$, and preferably at least $30^{m}$ [55]. Accordingly, in the present work the maximum scale factor was established to 40, thus entailing a minimum length of 768 samples for every coarse-grained time series. Moreover, for a wide characterization of complexity of the *f*-waves, profiles of MSE, CMSE and RMSE as a function of τ were globally parametrized as proposed in other studies [56,57]. Thus, these curves exhibited three clear regions, defined by low (τ=1−10), middle (τ=11−20) and high (τ=21−40) scales, and the area enclosed under each one was computed. The area for low scales was referred to as $A_{LS}$, for middle scales as $A_{MS}$, and for high scales as $A_{HS}$. Additionally, the curve for each region was also approximated as a first-degree polynomial using a minimum square adjustment method. The slope of the resulting line was referred to as $S_{LS}$ for low scales, $S_{MS}$ for middle scales, and $S_{HS}$ for high scales.

### 2.4. Performance Assessment

All described indices characterizing the *f*-waves were evaluated for normality by means of the Kolmogorov-Smirnov hypothesis test [58]. This assumes as null hypothesis that data come from a standard normal distribution, which was here rejected at a 5% of significance level. When data for patients maintaining SR and relapsing to AF came from normal distributions, a Student’s *t*-test was used to assess statistical separability between them [59]. Otherwise, a Wilcoxon rank sum test was used for that purpose [60]. In both cases, the null hypothesis was rejected with a significance level of 5%.

On the other hand, the discriminant ability of the analyzed parameters was studied by means of a receiver operating characteristic (ROC) curve. This plot provides information on how each variable can be used as a classifier by computing true positive (sensitivity) and false positive (1 − specificity) classification probabilities. The area under the ROC curve (AROC) is then an aggregate measure of performance of a variable across all possible classification thresholds. The ideal value of AROC is 1 and the worst-case value is 0.5 [61]. In the present study, sensitivity (Se) was considered as the proportion of patients who relapsed to AF, whereas specificity (Sp) was defined as the percentage of patients who maintained SR at the end of the follow-up. Moreover, the optimal threshold to discern between patients maintaining SR and relapsing to AF was computed according to the Youden’s criterion, i.e., by maximizing the addition of true positive and true negative ratios [62].

## 3. Results

### 3.1. Performance of Existing Predictors Analyzing the *f*-Waves

**Figure 2:**
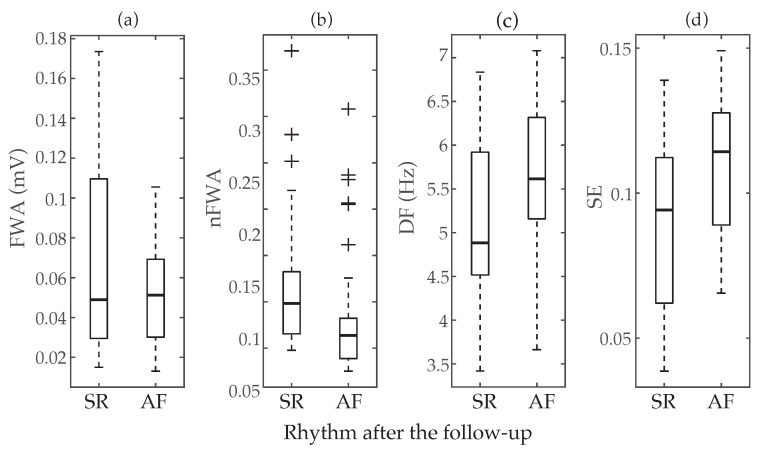
Boxplot distributions of the parameters previously proposed in the literature to predict ECV outcome, i.e., (**a**) *f*-waves amplitude (FWA), (**b**) normalized *f*-waves amplitude (nFWA) (**c**) dominant frequency (DF), and (**d**) Sample Entropy (SE). The central mark indicates the median, the bottom and top edges of the box indicate the 25th and 75th percentiles, respectively, and the whiskers extend to the most extreme data points not considered outliers. The outliers are plotted using the symbol +.

Median and interquartile ranges, along with classification results based on ROC curves, for the parameters previously proposed in the literature to anticipate early failure of ECV are presented in Table 2. Moreover, the boxplot distributions of these metrics for patients who maintained SR and relapsed to AF are also displayed in Figure 2. As it can be seen, FWA reported completely overlapped values for both groups, with median values of 0.051 and 0.050 mV for patients relapsing to AF and maintaining SR after the follow-up, respectively. Indeed, no statistically significant differences between groups were noticed (p=0.369) and poor values of AROC and Sp between 40 and 57% were also obtained. Contrarily, nFWA presented higher median values for patients maintaining SR than for those relapsing to AF (i.e., 0.098 vs. 0.064), with statistically significant separation between them (*p* < 0.05) and a discriminant ability above 72%. Only a slightly lower value of AROC about 70% was provided by DF, but in this case statistically significant larger median values of frequency were observed for patients who presented AF recurrence (5.615 Hz) than for those who maintained SR (4.883 Hz). A very similar result was also obtained by SE, where patients who maintained SR during the follow-up exhibited statistically significant shorter median values of entropy than those who relapsed to AF, i.e., 0.094 vs. 0.114. Nonetheless, in this case a slightly larger imbalance between Se and Sp than for DF was noticed, since they presented values about 72 and 58%, respectively.

### 3.2. Performance of Multi-Scale Entropy-Based Predictors Analyzing the *f*-Waves

**Figure 3:**
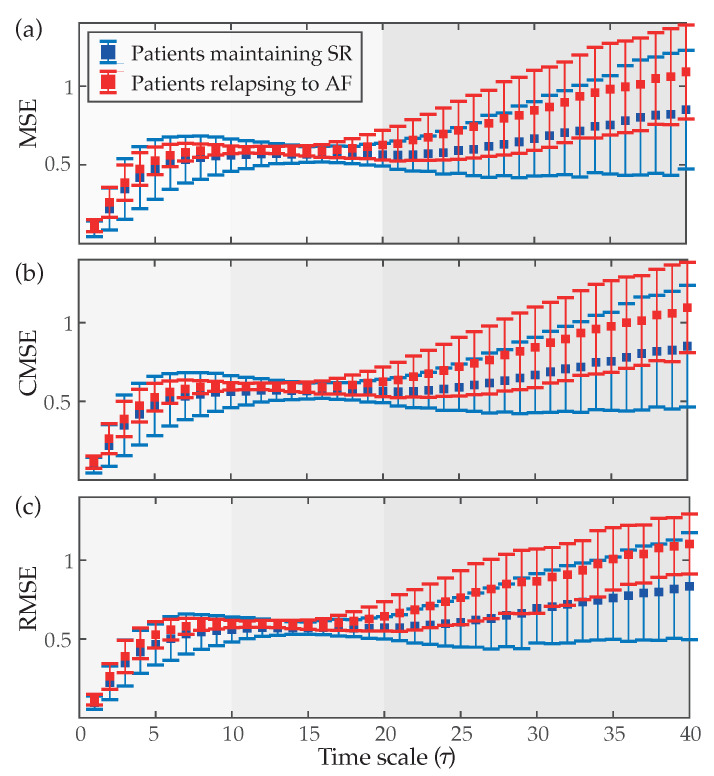
Median values (symbol ◼) and interquartile ranges (whiskers) of (**a**) multi-scale entropy (MSE), (**b**) composite MSE (CMSE) and (**c**) refined MSE (RMSE) as a function of the time scale (τ) for patients maintaining SR and relapsing to AF at the end of the follow-up.

Figure 3 displays how values of MSE, CMSE and RMSE evolve as a function of the scale factor τ. As can be seen, very similar curves were obtained for the three cases. Patients who relapsed to AF always presented values of entropy higher than those who maintained SR at the end of the follow-up. Nonetheless, as previously mentioned in Section 2.3.2, three clear regions can be observed in these curves. In the part defined by low scales (τ=1−10) entropy reported a monotonic increase for both groups of patients, existing statistically significant differences between them (p<0.05) for every time scale. Regarding the region covering middle scales (τ=11−20), a nearly stable behavior with little variance and separation in values of entropy for both groups of patients was observed. However, *p*-values lower than 0.05 were also noticed for these time scales. Finally, in the region comprising high scales (τ≥21), both groups of patients showed increasing and divergent trends in values of entropy. Thus, while a notably higher variance in estimates of entropy was observed for larger time scales, median values also tended to be more separated and statistically significant differences between groups were always observed. Moreover, values of AROC higher than 75% were observed for all time scales larger than 21, regardless of the multi-scale entropy approach.

**Figure 4:**
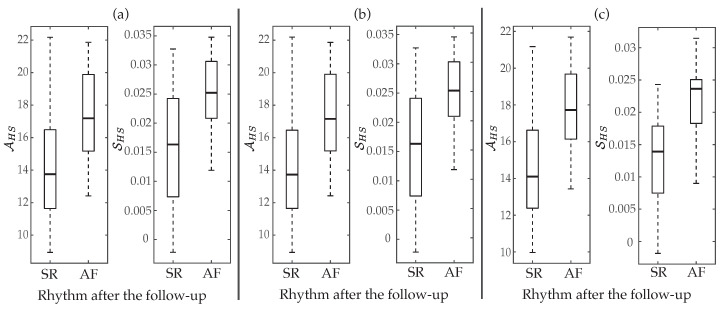
Boxplot distributions of $A_{HS}$ and $S_{HS}$ for (**a**) MSE, (**b**) CMSE, and (**c**) RMSE. The central mark indicates the median, the bottom and top edges of the box indicate the 25th and 75th percentiles, respectively, and the whiskers extend to the most extreme data points not considered outliers.

**Figure 5:**
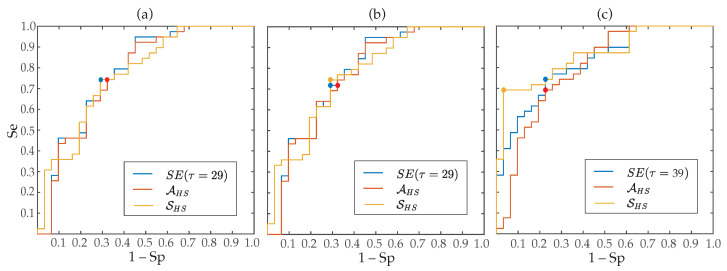
ROC curves for SE obtained from the best time scale, $A_{HS}$ and $S_{HS}$ derived from (**a**) MSE, (**b**) CMSE, (**c**) RMSE. The point marks optimal threshold according to the Youden’s criterion.

Regarding the aggregated complexity measure A, statistically significant differences between groups of patients (p<0.05) were noticed for the three regions delimited in the MSE curves, i.e., for LS, MS and HS, but $A_{HS}$ presented a value of AROC 2% higher than $A_{LS}$ and 8% higher than $A_{MS}$. Contrarily, the index S did not report statistical significance (p>0.05) for low and middle scales, only providing values of AROC below 63% for the three MSE approaches. However, although in HS entropy values exhibited the largest interquartile ranges, median values presented the highest separation between groups of patients, consequently $S_{HS}$ provided statistical significance p<0.05 and the highest ability to predict ECV outcome. More precisely, median values and interquartile ranges for the parameters $A_{HS}$ and $S_{HS}$, are presented in Table 3 and displayed in Figure 4, respectively. As it can be observed, for the three multi-scale approaches, larger values of $A_{HS}$ and $S_{HS}$ were provided for patients who relapsed to AF than for those who maintained SR. Moreover, their classification results were very similar to those reported by the best scale for MSE (scale τ=29), CMSE (scale τ=29) and RMSE (scale τ=39), such as Table 3 also presents. Nonetheless, it should be remarked that metrics computed from the RMSE curve performed about 3–9% better than those obtained from the MSE and CMSE profiles. In fact, the largest AROC of 86% was achieved by the index $S_{HS}$ calculated from the RMSE curve. While a significant imbalance between Se and Sp was reported by this metric, Figure 5 shows that changing the threshold to achieve Sp between 70 and 95% still provided Se above 70%, thus overcoming all remaining metrics.

## 4. Discussion

Previous studies have suggested that the heart’s behavior is far from being linear during AF, since a non-uniform and anisotropic atrial conduction occurs when the arrhythmia is present [63,64]. More precisely, it has also been pointed out that typical steep conduction velocity dispersion during AF represents one way of forming a spatially heterogeneous pattern in a completely homogeneous tissue [65]. Such a pattern formation has been described by theories dealing with nonlinear and highly complex systems [63,64]. To this respect, the application of nonlinear methods to *f*-waves has received great attention in the last years [66]. However, no time-scale analyses have been conducted yet, thus ignoring information from a wider characterization of complex fibrillatory dynamics [38]. To the best of our knowledge, the present study has introduced for the first time a multi-scale entropy analysis of *f*-waves to provide improved proarrhythmic condition estimation, thus anticipating early failure of ECV in patients suffering from persistent AF.

The obtained results precisely highlight that MSE, CMSE and RMSE have been able to gain additional insights compared to the single-scale analysis conducted with SE. In fact, entropy values estimated from most time scales, as well as parameters globally summarizing MSE, CMSE and RMSE curves, provided a discriminant ability between 3 and 15% better than SE (see Table 2 and Table 3). However, these improvements in classification depended on the analyzed time scales. More precisely, entropy estimated from low and middle time scales only presented values of AROC about 3–5% better than SE. This result could be explained by the fact that the original ECG recordings were initially acquired with a too large sampling rate with respect to the relevant frequency content of *f*-waves, thus leading to a high similarity between consecutive samples. Once this oversampling was reduced for middle scales, values of entropy remained approximately constant because spectral content of *f*-waves was mainly unaltered in the resulting coarse-grained time series. Finally, when fast oscillations in *f*-waves were removed, entropy began to increase for large time scales. To this respect, it should be noted that the coarse-grained time series for the scale τ=20 was sampled with a rate of about 50 Hz, for the scale τ=30 of about 35 Hz, and for the scale τ=40 of about 25 Hz. This filtering of high-frequency oscillations in successive time scales allowed to enhance fundamental information in *f*-waves, thus separating mean values of entropy for patients maintaining SR and relapsing to AF and then reaching a discriminant ability between 7 and 15% better than SE.

A similar three-way behavior in the profile of MSE has also been observed when alpha brain waves in electroencephalographic recordings were analyzed from patients with disorders of attention-deficit and hyperactivity [67]. Far beyond any other similarity between both kinds of signals, alpha brain waves and *f*-waves exhibit a similar frequency range [67]. Moreover, given that a similar oversampling to the present work was also considered in [67], the results obtained in both cases suggest that the most different dynamics could be seen among those time scales covering the spectral content associated with the most relevant information in the signal. According to this observation, areas and slope computed from low and middle scales in MSE, CMSE and RMSE profiles were much less predictive of ECV outcome than those obtained from large scales. Moreover, times scales providing the highest classification results were also located in the remarked third region, i.e., τ=29 both for MSE and CMSE, and τ=39 for RMSE (see Table 3).

Nonetheless, it is worth noting that patients who relapsed to AF presented higher values of entropy than those who maintained SR for all time scales (see Figure 3). This finding agrees with values of SE obtained in the present study as well as those presented by previous works [34,37], and suggests that the presence of more disorganized *f*-waves increases the probability of AF recurrence after ECV [34,37]. While the mechanisms supporting AF are still not fully understood [68], more irregular *f*-waves could be indicative of more heterogeneity in atrial conduction, which could be associated with more advanced modification of structural and electrophysiological parameters of the atria, thus increasing the patient proarrhythmic condition [69,70]. In fact, the morphology of the *f*-waves on the surface ECG has been suggested to result from an interplay between viable atrial muscle mass and variability in atrial conduction [24,71]. Moreover, previous works have also found more disorganized *f*-waves in chronic than in initial stages of the arrhythmia [72].

On the other hand, no remarkable differences among MSE, CMSE and RMSE curves were observed (see Figure 3). Indeed, MSE and CMSE profiles were almost identical, and parameters derived from them also provided very similar classification outcomes (see Table 3). While CMSE was proposed to reduce variance in entropy estimates from large scales [40], its effect was only marginally seen in patients who relapsed to AF during the follow-up. The fact that all analyzed coarse-grained time series presented a large number of at least 768 samples could explain this outcome, thus suggesting that this modification of MSE could only play a more relevant role in the analysis of shorter signals. Contrarily, a notably higher reduction of variance in RMSE measures was noticed in large scales, especially for patients who relapsed to AF (see Figure 3). As a consequence, removal of aliasing artifacts, as well as reduction of artificial regularity by normalizing the threshold *r* to the standard deviation of the coarse-grained times series [41], seems to be essential for a better estimation of the organization of the *f*-waves. According to this finding, parameters based on the RMSE curve provided values of AROC between 3 and 9% higher than those obtained from the MSE and CMSE profiles.

Moreover, the best classification result obtained in the present work was reported by the parameter $S_{HS}$ also computed from the RMSE curve. In fact, this parameter presented an AROC of 86% (see Table 3). While highly unbalanced values of Se and Sp were noticed, when a different threshold from the Youden’s criterion was used to get Sp between 70 and 95%, better values of Se (above 70%) than all remaining predictors of ECV outcome were still observed (see Figure 5). This good result could be due to the fact that $S_{HS}$ is able to reflect the degree of change in entropy estimates over several time scales, thus providing a more global and accurate measure of structural complexity of *f*-waves. In fact, it is well-known that there exist differences between mathematical concepts of regularity and complexity, such that an increase in single-scale entropy mandatorily involves a loss of regularity but may not always be related to an increase in dynamical complexity [38]. Hence, having in mind that complexity has been associated with “meaningful structural richness” [73], $S_{HS}$ could be considered as a more robust complexity measure of *f*-waves than single-scale entropies. The same idea also applies for the metric $A_{HS}$, which globally summarizes entropy for several time scales. Thus, a higher area can only be achieved when entropy values are greater for most scales, then suggesting more complex time series. Consequently, this parameter for the three multi-scale approaches has provided discriminant abilities higher than single values of entropy obtained from most factors τ, and comparable to those reported by the best time scales (see Table 3).

All parameters derived from the MSE, CMSE and RMSE profiles also proved to be more predictive of ECV outcome than the indices previously proposed in the literature. Comparing Table 2 and Table 3, improvements about 7–16% in values of AROC were seen for multi-scale measures. Nonetheless, it should be noted that the results provided by FWA, nFWA, and DF were totally consistent with those reported in previous works. Thus, whereas FWA showed values mostly overlapped for both groups of patients and a poor discriminant ability [24], its normalized version nFWA obtained much more information. Indeed, this index obtained a discriminant ability about 70%, also exhibiting larger values for patients who maintained SR [37]. The differences observed for both indices agree with previous studies on the idea that expressing *f*-wave amplitude as a percentage of the R-peak magnitude is essential to reduce confounding effects of physiological variations among patients (e.g., chest wall attenuation, skin impedance, etc.) [35,37,74]. Moreover, in line with previous results [35,36,37], statistically significant larger values of DF were reported for patients who relapsed to AF than for those who maintained SR after the follow-up. Moreover, this index also provided a discriminant ability about 70% with well-balanced values of Se and Sp [35,37].

Finally, some limitations of the present study should be mentioned. First, seventy patients from a single centre were only studied. While the obtained results provided consistent trends in the MSE, CMSE and RMSE curves for both groups of patients, as well as statistically significant differences between them for most parameters, further prospective studies with larger databases should be warranted to confirm the relevant role of multi-scale entropy analysis of *f*-waves in estimating individual proarrhythmic conditions and anticipating early failure of ECV. Second, continuous heart rhythm monitoring of the patients within the whole follow-up was not possible, and therefore some self-limiting, asymptomatic episodes of AF recurrence could have been missed. Finally, lead $V_{1}$ was only analyzed, thus ignoring the possible information contained in the remaining signals. However, this lead has been suggested as the most suitable for the analysis of *f*-waves, because significant correlation between frequency [75], amplitude [76] and SE [76] obtained from this lead and from intra-atrial electrograms had been reported, and therefore it could reflect the global activation of the atria [75,76,77]. In fact, many previous works have only analyzed lead $V_{1}$ to anticipate ECV outcome [24,31,32,33,34,35,37,49,78]. Nonetheless, because DF analysis has provided a dissimilar predictive ability when applied to different channels of the standard ECG [36], the multi-scale entropy analysis conducted in the present work will be extended to multiple leads in future investigations.

## 5. Conclusions

The present work has introduced for the first time a multi-scale entropy analysis of the *f*-waves to evaluate the individual proarrhythmic condition, thus anticipating early failure of ECV in persistent AF patients. The obtained results have shown a different behavior in the values of entropy for low, middle, and high time scales, revealing most predictive information from those covering frequency bands close to the spectral content of interest in the *f*-waves (i.e., time scales larger than 20). Indeed, single entropy values obtained for large time scales achieved a discriminant ability between 4 and 10% greater than for short scales, while providing better trade-off between sensitivity and specificity. Moreover, global characterization of the dynamics exhibited by these large scales has also reported more robust estimates of complexity of the *f*-waves than the values of entropy computed from single time scales, thus improving classification between patients who relapsed to AF and maintained SR after the follow-up between 3 and 14%. Moreover, the best classification result was obtained by the slope estimated from the RMSE curve for high scales. This index has reported an AROC of 86%, thus improving between 3 and 9% the discriminant ability of the remaining multi-scale-based parameters, and between 15 and 30% that of other previously proposed predictors of ECV outcome. In view of these results, the multi-scale entropy analysis of the *f*-waves, and especially the quantification of the entropy change over large time scales, could be extremely helpful in clinical decisions regarding optimal management of patients with persistent AF. Nonetheless, further prospective studies with larger databases are required to validate the robustness of the obtained results.

## Figures and Tables

**Table 1:** Clinical characteristics for the population under study.

Features	Group of Patients	
	Maintaining SR	Relapsing to AF
Patients	31	39
Men/Women	15/16	17/22
Underlying heart disease	10 (32.26%)	11 (28.21%)
Left atrial diameter (mm)	44.24 ± 7.53	47.02 ± 5.37

**Table 2:** Median and interquartile ranges for the metrics previously proposed to predict ECV outcome. Statistical significance (*p*-value) between groups of patients relapsing to AF and maintaining SR, as well as classification results, are also provided for each index.

Parameter	Group of Patients		*p*-Value	AROC	Se	Sp
	Relapsing to AF	Maintaining SR				
FWA (mV)	0.051 (0.036)	0.050 (0.036)	0.369	0.565	0.846	0.419
nFWA	0.064 (0.043)	0.098 (0.067)	0.001	0.724	0.718	0.613
DF (Hz)	5.615 (1.160)	4.883 (1.404)	0.003	0.707	0.692	0.613
SE	0.114 (0.039)	0.094 (0.050)	0.004	0.704	0.718	0.581

**Table 3:** Median and interquartile ranges for the metrics computed from the three analyzed multi-scale approaches. Statistical significance (*p*-value) between groups of patients relapsing to AF and maintaining SR, as well as classification results, are also provided for each index.

Approach	Parameter	Group of Patients		*p*-Value	AROC	Se	Sp
		Relapsing to AF	Maintaining SR				
	SE(τ = 29)	0.814 (0.242)	0.646 (0.228)	<0.001	0.777	0.744	0.710
MSE	$A_{HS}$	17.189 (4.716)	13.747 (4.841)	<0.001	0.767	0.744	0.677
	$S_{HS}$	0.025 (0.010)	0.016 (0.017)	<0.001	0.768	0.744	0.710
	SE(τ = 29)	0.818 (0.228)	0.649 (0.243)	<0.001	0.773	0.718	0.710
CMSE	$A_{HS}$	17.158 (4.721)	13.728 (4.817)	<0.001	0.768	0.744	0.678
	$S_{HS}$	0.025 (0.009)	0.016 (0.017)	<0.001	0.771	0.744	0.710
	SE(τ=39)	1.059 (0.307)	0.825 (0.374)	<0.001	0.825	0.744	0.774
RMSE	$A_{HS}$	17.727 (3.538)	14.104 (4.243)	<0.001	0.794	0.692	0.774
	$S_{HS}$	0.024 (0.007)	0.014 (0.010)	<0.001	0.860	0.692	0.968
